# L'atteinte osseuse dans le sarcome de Kaposi classique et agressif: à propos d'un cas

**DOI:** 10.11604/pamj.2016.23.196.8632

**Published:** 2016-04-15

**Authors:** Mouhcine Sbiyaa, Adil El Alaoui, Mohammed El Bardai, Amine Mezzani, Kamal Lahrach, Amine Marzouki, Fawzi Boutayeb

**Affiliations:** 1 Service de Chirurgie Orthopédique et Traumatologique (A), Centre Hospitalier Universitaire Hassan II de Fès, Maroc

**Keywords:** Sarcome, Kaposi, agressif, Sarcoma, Kaposi, aggressive

## Abstract

Le sarcome de Kaposi classique est une tumeur rare multifocale d'origine des cellules endothéliales vasculaires à caractère évolutif progressif et peu maligne. L'atteinte viscérale dans le sarcome de kaposi est parfois observée chez les patients VIH positif par contre la dissémination tumorale dans les ganglions lymphatiques viscérales dans le SK classique reste très rare. On rapporte un cas rare de sarcome de kaposi classique agressif de la main avec une évolution rapide et destructive.

## Abstract (en)

Classic Kaposi sarcoma is a multifocal rare tumor originating from vascular endothelial cells with progressive evolution and little malignant predisposition. Although Kaposi sarcoma with extensive visceral involvement is sometimes observed among HIV-positive patients, tumor dissemination to visceral lymph nodes in classic SK remains very rare. We report a rare case of aggressive classic Kaposi sarcoma of the hand with a rapid and destructive development.

## Introduction

Le sarcome de Kaposi (SK) est une tumeur rare décrite par Moritz Kaposi comme une maladie bénigne de personnes âgées [1]. Quatre formes cliniques sont actuellement reconnues: SK classiques, SK endémiques africains, SK iatrogène lié à la transplantation et SK épidémique associée au SIDA. Bien que les quatre types aient différentes formes d′évolution, ils ont des caractéristiques phénotypiques similaires [2]. Cette tumeur est lentement progressive. Elle est caractérisée par l′apparition de macules mauves sur la partie distale des membres qui peuvent acquérir un caractère tumorales nodulaires. La diffusion aux organes internes peut se produire dans les formes les plus avancées [3, 4]. On rapporte un cas rare de SK classique agressive de la main avec une évolution rapide et destructive.

## Patient et observation

**Figure 1:**
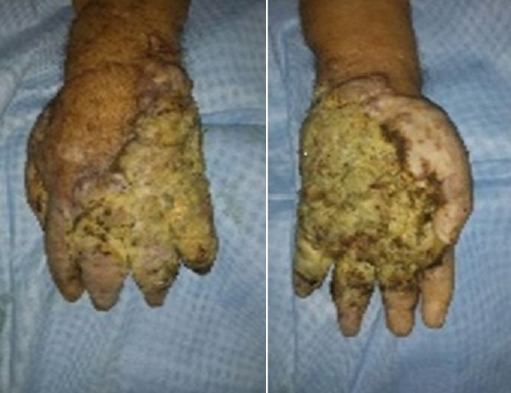
Les lésions du sarcome de Kaposi sur la main: papules, des nodules, des lésions tumorales et des plaques d'infiltration. Exsudation Importante, sécrétions purulentes et des zones de nécrose

**Figure 2:**
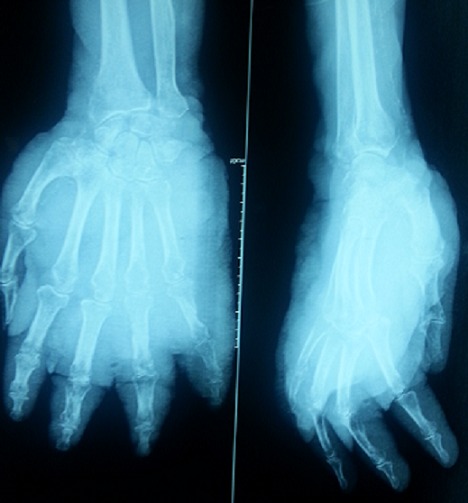
Radiographie de la main face et profil montrant l'atteinte osseuse

**Figure 3:**
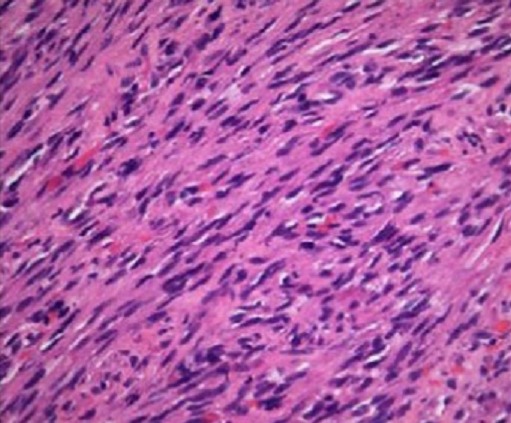
Coupe histologique: Faisceaux des cellules fasciculées relativement monomorphes avec des fentes vasculaires contenant des érythrocytes

nous rapportons le cas d′un patient âgé de 100 ans sans antécédent pathologiques notables qui a consulté pour des lésions pigmentées de la main gauche qui remonte à 20 ans, compliqué depuis 2 mois de douleur avec un saignement minime. L'examen clinique a révélé un placard kératosique surmonté de nodules angiomateuses avec le lymphœdème de la main gauche, la sérologie VIH était négative et la biopsie cutanée était en faveur d′un SK nodulaire. La décision de radiothérapie a été posée, mais le patient a refusé le suivi. Après un mois, il a consulté aux urgences pour un hyper bourgeonnement nodulaire associé à une douleur atroce, nécrose partielle et une ulcération des doigts (Figure 1). La radiographie standard de la main gauche a objectivé une destruction osseuse massive de la main (Figure 2). La décision d′une amputation trans antébrachiale a été prise. L′étude histologique et immunohistochimique de la pièce opératoire était en faveur de SK sans atypie cellulaire et seulement quelques images de mitose (Figure 3). L′examen général n′a pas objectivé de localisation secondaire.

## Discussion

SK classique est une maladie rare qui a un caractère évolutif lent et bénin. Elle touche principalement les hommes entre 40-70 ans [5, 6]. Son incidence varie selon les régions avec une fréquence plus élevé en Italie, la Grèce, la Turquie et Israël. Les lésions cutanées débutent généralement par l′apparition de macules pourpres dans la partie distale du membre qui évoluent très lentement (au cours des années, voir des décennies) vers des nodules, des plaques et des lésions tumorales. Le caractère multi focale du SK classique explique l′apparition de nouvelles lésions sur d′autres sites. L′évolution est marqué par le durcissement des lésions, devenant brune-coloré avec une surface irrégulière ulcéré et un œdème péri-lésionnel. La muqueuse est affectée chez environ 15% des cas et la forme viscérale de la maladie touche le plus souvent les ganglions lymphatiques et le tube digestif [7]. Toutefois, le foie, les poumons et le cœur peuvent également être affectés, ainsi que d′autres organes.

Les caractéristiques anatomo-pathologiques du SK sont une prolifération de cellules fusiformes et de cellules endothéliales associée à une extravasation de globules rouges, macrophages et d′infiltrat de cellules inflammatoires. L′atteinte osseuse contrairement aux lésions cutanées est connue comme une lyse ou une condensation osseuse, mais il n′a jamais été rapporté la destruction massive que l′on trouve chez notre patient [8, 9]. L′évolution rapide et agressive est principalement l′apanage des formes épidémiques et endémiques tandis que la forme classique évolue progressivement sur plusieurs années avec la possibilité de la stabilisation de l’évolution des lésions cutanées [10, 11].

La réponse de SK aux différentes stratégies thérapeutique est bonne [12]. Pour les lésions localisées, l'excision chirurgicale, la cryothérapie et la radiothérapie peuvent être utilisés. Pour des lésions cutanées plus importantes, des lésions multiples ou ceux affectant les organes internes, la thérapie systémique peut être indiqué. La chimiothérapie est utilisé dans les formes diffuses de la maladie, elle peut être efficace à la fois sur la peau et les lésions viscérales. Il y a divers agents chimiothérapeutiques actifs avec un taux de réponse de l′ordre de 60-80%. Chez notre patient, l′évolution était non seulement rapide, mais associée à un hyper bourgeonnement avec nécrose distale et une ulcération faisant évoquer plutôt un angio-sarcome qu′une dégénérescence du sarcome de kaposi. Devant ce comportement agressif plutôt inhabituel et aiguë de la maladie, il y avait aucune chance d′établir un traitement systémique pour préserver le membre affecté.

## Conclusion

L′atteinte osseuse a été retrouvée chez 4,5% des patients atteints d′un SK classiques [13]. Tous les sous-types du SK peuvent atteindre le système squelettique. Nous recommandons une évaluation approfondie de lésions osseuses chez les patients qui ont un SK, avec tumeurs localisées ou agressif. Un scanner ou une IRM devrait être inclus dans le bilan de ces patients, car une radiographie simple et la scintigraphie osseuse peuvent ne pas détecter une lésion osseuse. La biopsie est toujours nécessaire pour confirmer le diagnostic.
